# Crossmodal deficit in dyslexic children: practice affects the neural timing of letter-speech sound integration

**DOI:** 10.3389/fnhum.2015.00369

**Published:** 2015-06-24

**Authors:** Gojko Žarić, Gorka Fraga González, Jurgen Tijms, Maurits W. van der Molen, Leo Blomert, Milene Bonte

**Affiliations:** ^1^Department of Cognitive Neuroscience, Faculty of Psychology and Neuroscience, Maastricht UniversityMaastricht, Netherlands; ^2^Maastricht Brain Imaging Center (M-BIC)Maastricht, Netherlands; ^3^Department of Developmental Psychology, University of AmsterdamAmsterdam, Netherlands; ^4^Rudolf Berlin CenterAmsterdam, Netherlands; ^5^IWAL InstituteAmsterdam, Netherlands; ^6^Amsterdam Brain and Cognition, University of AmsterdamNetherlands

**Keywords:** developmental dyslexia, cross-modal integration, mismatch negativity, ERP, training effects

## Abstract

A failure to build solid letter-speech sound associations may contribute to reading impairments in developmental dyslexia. Whether this reduced neural integration of letters and speech sounds changes over time within individual children and how this relates to behavioral gains in reading skills remains unknown. In this research, we examined changes in event-related potential (ERP) measures of letter-speech sound integration over a 6-month period during which 9-year-old dyslexic readers (*n* = 17) followed a training in letter-speech sound coupling next to their regular reading curriculum. We presented the Dutch spoken vowels /a/ and /o/ as standard and deviant stimuli in one auditory and two audiovisual oddball conditions. In one audiovisual condition (AV0), the letter “a” was presented simultaneously with the vowels, while in the other (AV200) it was preceding vowel onset for 200 ms. Prior to the training (T1), dyslexic readers showed the expected pattern of typical auditory mismatch responses, together with the absence of letter-speech sound effects in a late negativity (LN) window. After the training (T2), our results showed earlier (and enhanced) crossmodal effects in the LN window. Most interestingly, earlier LN latency at T2 was significantly related to higher behavioral accuracy in letter-speech sound coupling. On a more general level, the timing of the earlier mismatch negativity (MMN) in the simultaneous condition (AV0) measured at T1, significantly related to reading fluency at both T1 and T2 as well as with reading gains. Our findings suggest that the reduced neural integration of letters and speech sounds in dyslexic children may show moderate improvement with reading instruction and training and that behavioral improvements relate especially to individual differences in the timing of this neural integration.

## Introduction

Although most children learn to read fluently, between 5 and 10% of children are diagnosed with developmental dyslexia exhibiting deficient reading skills despite normal cognitive abilities and schooling opportunities (Lyon et al., 2003; Blomert, 2005; Snowling, 2013). The formation of letter-speech sound pairs, an important first step in obtaining reading expertise in alphabetic orthographies, forms an immediate obstacle for beginner readers with dyslexia (Share, 1995; Ehri, 2005; Wimmer and Schurz, 2010; Blomert, 2011). Consequently, many dyslexia interventions include modules focused on teaching letter-speech sound correspondences (Bus and Van Ijzendoorn, 1999; Tijms and Hoeks, 2005; Aravena et al., 2013) next to dealing with impaired phonological processing (Snowling, 1998; Ramus and Szenkovits, 2008). Accumulating evidence from neuroimaging studies suggests reduced neural integration of letters and speech sounds in dyslexic children (Blau et al., 2010; Froyen et al., 2011; McNorgan et al., 2013; Žarić et al., 2014) and adults (Blau et al., 2009). However a remaining open question is whether an increased exposure to literacy and especially learning letter-speech sound correspondences can change the neural integration deficit in dyslexia.

In transparent orthographies such as Dutch, children typically learn to accurately identify and discriminate letter-speech sound pairs during the first year of reading instruction (Blomert and Vaessen, 2009). This is contrasted by the results of neuroimaging studies in which development of automatic neural integration shows a much more protracted period throughout primary school (Booth et al., 2001; Froyen et al., 2009). This prolonged maturation of the neural integration of letters and speech sounds has been observed in studies measuring electroencephalogram (EEG) responses in a passive crossmodal “oddball” paradigm (Froyen et al., 2008, 2009, 2011; Žarić et al., 2014). In oddball paradigms a mismatch negativity (MMN) response is elicited at 100–250 ms after presentation of a so called oddball or deviant sound within a train of frequent (standard) sounds. The MMN is believed to reflect an automatic response to deviation from traces formed in auditory short-term memory due to the frequent repetition of the standard stimulus (Näätänen, 2001; Näätänen et al., 2007). The MMN response is sensitive to both language-specific speech sounds and audiovisual integration in children and adults (Cheour-Luhtanen et al., 1995; Csépe, 1995; Näätänen, 2001; Cheour et al., 2002; Kasai et al., 2002; Bonte et al., 2007; Froyen et al., 2008, 2009; Andres et al., 2011; Mittag et al., 2011, 2013; Neuhoff et al., 2012; Lohvansuu et al., 2013; Žarić et al., 2014). Additionally, a broader late negativity (Late MMN or LN), between 300–700 ms after deviant onset, can be seen in school-aged children, while it is diminished in adults (Cheour et al., 2001; Froyen et al., 2008; Hommet et al., 2009; Czamara et al., 2011), suggesting the recruitment of additional processing resources in children.

**Figure 1:**
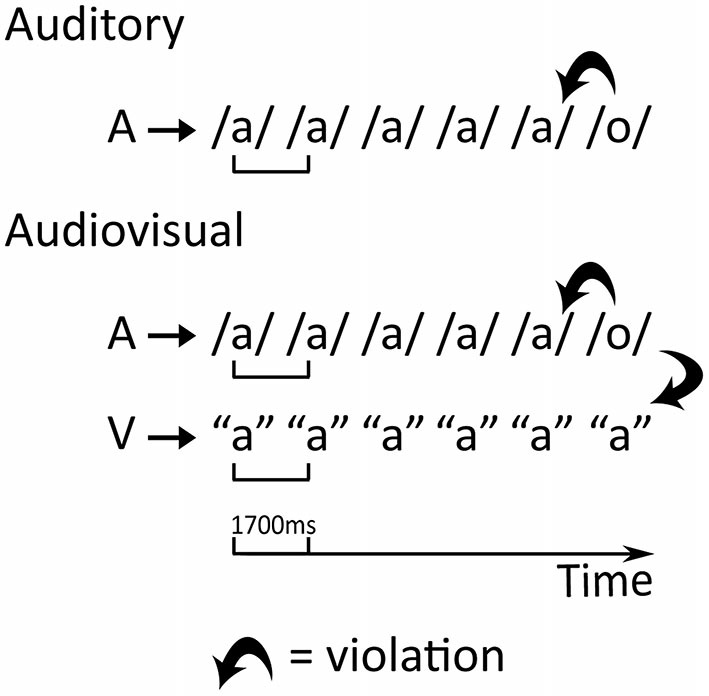
**Design of the auditory and the audiovisual conditions.** A—auditory stimulus, V—visual stimulus. The thick bent arrows represent the auditory mismatch in the auditory condition and the double, auditory and visual, mismatch in the audiovisual conditions.

The crossmodal oddball paradigm (Froyen et al., 2008, 2009, 2011; Žarić et al., 2014) consists of an auditory and two audiovisual experimental conditions during which the Dutch speech sounds /a/ and /o/ are presented as the standard and deviant stimuli respectively. In the audiovisual blocks, the letter “a” is paired with the speech sounds resulting in a double mismatch: the deviant speech sound /o/ is incongruent both with respect to the standard speech sound /a/ and the presented letter “a” (Figure 1). The stimulus onset asynchrony (SOA) between the letter and the speech sound is either 0 or 200 ms to allow investigation of the temporal window of integration. In adults, this paradigm showed an early MMN enhancement due to the presentation of the letter and only with synchronous letter-speech sound pairs (Froyen et al., 2008), while children additionally showed later LN letter effects and a developmental shift in the temporal integration window (Froyen et al., 2009; Žarić et al., 2014). In agreement with an inverted “U” trajectory of increased brain responses during early school years followed by reduced and more selective responses with age and experience (Bonte and Blomert, 2004; Maurer et al., 2006), the MMN and LN of 9-year-old typical readers exhibited stronger crossmodal effects than in younger (8-year-old) as well as older (11-year-old) readers and adults. In particular, 9-year-old children showed enhanced crossmodal MMN and LN responses to both synchronous and asynchronous letter-speech sound pairs (Žarić et al., 2014). Furthermore, although adults did not show the later LN in this passive paradigm using simple speech stimuli (Froyen et al., 2008), they have been reported to show late orthographic-phonological interactions in spoken language processing (400–700 ms) during more complex metaphonological tasks (Pattamadilok et al., 2011; Lafontaine et al., 2012). It can thus be speculated that while the crossmodal MMN enhancement reflects early and automatic letter-speech sound integration and/or representation (Näätänen, 2001; Näätänen et al., 2007), the crossmodal LN enhancement reflects more elaborate associative processes that are recruited for the integration of simple letter-speech pairs during initial learning phases, but become redundant once this integration becomes automatic and overlearned.

In dyslexic children, the crossmodal oddball paradigm indicated typical vowel-evoked change detection responses together with reduced crossmodal effects (Froyen et al., 2011; Žarić et al., 2014). In particular, all 9-year-old dyslexic participants showed a reduction in the crossmodal LN effect independently of the synchronicity of the letter-speech sound pairs, while the MMN response to synchronous letter-speech sound pairs was reduced only in the most severely dysfluent dyslexics (Žarić et al., 2014). Moreover, MMN latency in the synchronous condition was coupled with individual differences in reading fluency (Žarić et al., 2014). Further evidence comes from fMRI studies in 9-year-old children and adults with dyslexia demonstrating reduced neural integration of letters and speech sounds in superior temporal cortical regions (Blau et al., 2009, 2010). EEG studies in dyslexic adults additionally reported the absence of a crossmodal MMN enhancement for spoken-written syllable pairs (Mittag et al., 2013) as well as reduced orthographic-phonological integration during word reading tasks (Savill and Thierry, 2011, 2012; Hasko et al., 2012, 2013). Interestingly, a recent behavioral study (Aravena et al., 2013) employing an artificial script for letter-speech sound learning showed that children with dyslexia reached comparable levels of letter-speech sound knowledge to their typical reading peers, while remaining less fluent in mapping of letter-speech sound pairs. Thus, knowledge of letter-speech sound pairs by itself is not sufficient for the automation of letter-speech sound integration, and dyslexics may exhibit a specific deficit in the automation of this integration.

Here we employ the passive crossmodal oddball paradigm (Froyen et al., 2008, 2009, 2011; Žarić et al., 2014) to investigate whether 9-year-old dyslexic children exhibit changes in crossmodal EEG responses over time and whether these changes relate to behavioral improvements in reading fluency and letter-speech sound coupling. To this end, we examine MMN and LN measures of letter-speech sound integration in the same children after 2, 5 (T1) and 3 years (T2) of reading instruction, spanning a 6 months period during which they followed the first part of a systematic cognitive reading intervention program focusing on letter-speech sound integration (Tijms, 2004, 2007, 2011). The 17 children that were followed in the present study are a subgroup of the 36 dyslexic children that participated in a previous study (Žarić et al., 2014) and includes those that came back for EEG and behavioral measurements at T2. As we collected EEG and behavioral data at both measurements, we examine the relation between event-related potential (ERP) and behavioral measures at T1 based on our previous study (Žarić et al., 2014), as well as the predictive power of these ERP measures with respect to behavioral change, and the relation between changes in ERP and behavioral measures following the training.

## Methods

### Participants

In total, 19 dyslexic children participated in a first (T1) and second (T2) EEG session after 6 months. All children were native Dutch speakers, with 2.5 years of reading instruction at T1 and 3 years of reading instruction, including 34 sessions of reading intervention (Tijms, 2004, 2007, 2011) at T2. Data of 17 children were included in the analysis (on average 9.0 years old at T1, range: 8.2–9.9 years; 12 girls) Three children were left-handed, as assessed with a modified version of Annett’s handedness questionnaire (Annett, 1979). Data of 2 children were discarded, one due to the malfunction of recording reference electrodes and the other due to excessive movements during the first EEG measurement.

All children included in the study were diagnosed as dyslexic after an extensive cognitive psycho-diagnostic procedure at a specialized institute for dyslexia and reading problems (IWAL institute Amsterdam) and scored lower than the 10th percentile of the age appropriate group on standard reading tests (see below). Other behavioral scores such as phonological skills or rapid automatized naming (RAN) were not used as selection criteria for the current study. Parents reported normal hearing and normal or corrected to normal vision for all children. Parents also completed the Child Behavior Checklist (CBCL), a part of the Achenbach system of empirically based assessment (ASEBA; Achenbach and McConaughy, 2003). Exclusion criteria included comorbidity with behavioral and/or attention disorders (assessed with the CBCL) and below average IQ. Written informed consent was obtained from the parents of each child. We reimbursed travel costs and each child was given a small present after each session of the experiment. The approval for the research was obtained from the ethical committee of the Developmental Psychology Department of the University of Amsterdam (Ethics file 2011-OP-1907) and the research was conducted according to the relevant regulatory standards.

### Behavioral Tests

Preceding the first EEG measurement, children underwent individual testing on standard language tests including the one-minute word reading test (Eén Minuut Test (EMT); Brus and Voeten, 1973), and reading of a short story “De kat” (“The cat”; de Vos, 2007) as well as subtests of the Dyslexia Differential Diagnosis 3DM battery (Blomert and Vaessen, 2009), assessing word reading, spelling, letter-speech sound identification, letter-speech sound discrimination, rapid automized naming (RAN) and basic reaction time. Additionally, we assessed non-verbal IQ scores by means of a paper and pencil version of the RAVEN Coloured Progressive Matrices (RAVEN CPM; Raven et al., 1998). At the time of the second EEG measurement children performed a shorter version of the behavioral test battery including the word reading, spelling, letter-speech sound identification and letter-speech sound discrimination subtests of the 3DM battery, EMT and “De kat”. Subject characteristics and the results of the behavioral tests are shown in Table 1. For each test that was completed at both measurements, differences between the two measurements were tested using repeated measures ANOVA (see “Results” Section).

**Table 1:** **Descriptive statistics showing reading accuracy and fluency scores**.

	Standard scores*							Raw scores**						
	Pretest		Posttest					Pretest		Posttest				
	M	SD	M	SD	F_(1,16)_	*P*	η^2^	M	SD	M	SD	F_(1,16)_	*P*	η^2^
**Word reading—accuracy**
3DM Total Word Accuracy	**33.06**	**12.76**	**40.71**	**14.54**	**6.20**	**0.024**	**0.279**	**83.79**	**11.26**	**90.15**	**11.75**	**6.48**	**0.022**	**0.288**
**Word reading—fluency**
3DM Total Word Fluency	**29.41**	**5.40**	**33.06**	**6.42**	**21.05**	**0.000**	**0.568**	**54.71**	**14.09**	**72.65**	**17.67**	**89.99**	**0.000**	**0.849**
One minute test—EMT	3.29	1.76	3.71	2.23	1.05	0.322	0.061	**25.88**	**7.50**	**32.94**	**8.57**	**22.48**	**0.000**	**0.584**
**Text reading—fluency**
‘De Kat’	32.59	5.36	33.88	5.73	3.05	0.100	0.160	**75.76**	**18.16**	**89.24**	**18.22**	**28.46**	**0.000**	**0.640**
**Letter –speech sound coupling**
3DM Spelling—accuracy	**35.29**	**7.82**	**43.12**	**8.62**	**16.19**	**0.001**	**0.503**	**59.37**	**11.16**	**74.19**	**11.06**	**30.59**	**0.000**	**0.657**
3DM Spelling—RT	41.53	7.56	45.59	10.74	3.85	0.067	0.194	**4.22**	**0.77**	**3.57**	**0.79**	**24.57**	**0.000**	**0.606**
L-SS identification—accuracy	43.06	13.25	45.29	8.43	0.43	0.521	0.026	90.46	7.89	92.81	3.89	1.38	0.257	0.079
L-SS discrimination—accuracy	45.53	8.81	47.18	9.99	0.72	0.410	0.043	86.14	6.19	87.84	7.46	1.37	0.259	0.079
L-SS identification—RT	46.82	6.33	49.71	10.77	1.57	0.229	0.089	**2.26**	**0.29**	**2.05**	**0.40**	**6.36**	**0.023**	**0.284**
L-SS discrimination—RT	53.12	7.28	56.53	8.54	2.69	0.121	0.144	**1.50**	**0.26**	**1.32**	**0.26**	**7.07**	**0.017**	**0.306**

*T scores (M = 50, SD = 10) for all tests except EMT with Standard scores (M = 10, SD = 3). **All accuracy measures [% correct]; 3DM Total Word Fluency [N words /30 sec]; EMT and “De Kat” [N words/60 sec]; All RT measures [sec/item].

### Reading Instruction and Training

Next to their regular reading curriculum at school, all children took part in a training program (Tijms, 2004, 2007, 2011) that was provided twice per week on a one-to-one basis with each session lasting 45 min. Training was performed with a computer-assisted training program and guided by a tutor. Sessions consisted of an instruction and a practice part. First, letter-speech sound correspondences were explicitly trained during the instruction part, with the aim to achieve a mastery level, i.e., at least 80% of the items correctly executed at each step. Second, during the practice part, a high exposure to the trained letter-speech sound associations was provided to stimulate the automatic integration of letters and speech sounds. The letter-speech sound correspondences were taught in a step-by-step fashion, increasing the level of complexity, e.g., from the short vowels to the diphthongs and starting with the regular letter-speech sound correspondences before continuing with the irregular ones. The computer training progressed within time constraints, which were adapted to the individual performance of each child.

### Stimuli

The stimuli and experimental design were the same as in our previous study (Žarić et al., 2014) involving digital recordings of a native Dutch female speaker pronouncing the Dutch vowels /a/ and /o/ (sampling rate 44.1 kHz, 16 bit quantization; band-pass filtered: 180–10,000 Hz; downsampled to 22.05 kHz and matched for loudness using Praat software; Boersma and Weenink, 2002). Sound duration was 384 ms for vowel /a/ and 348 ms for vowel /o/. The vowel sounds were presented to both ears via headphones with a loudness of ~65 dB as measured with an analog loudness meter. The letter stimulus consisted of a white “a” presented in lower case “Arial” font, size 40, in the center of a black computer screen. Presentation 14.4 (Neurobehavioral Systems, Inc., Albany, CA, USA) was used for stimulus presentation.

### Experimental Design

The standard stimulus, vowel /a/, was presented in 83% of trials while the deviant stimulus, vowel /o/, was presented in 17% of the trials, in one auditory and two audiovisual oddball conditions (Figure 1). In the two audiovisual conditions, the letter “a” was presented together with the vowels for 500 ms. Between successive letter presentations, a white fixation cross appeared in the same location. Trial length was always 1700 ms. The difference between the two audiovisual conditions involved the SOA between the presentation of the letter and vowel stimuli. In the synchronous audiovisual condition (AV0), the letter and vowel appeared simultaneously, while the letter appeared 200 ms before the vowel in the asynchronous audiovisual condition (AV200). To ensure that participants fixated on the screen during the audiovisual conditions, we employed a simple visual target detection task in which they had to press a button when a target (color picture of a wrapped present) was presented instead of the letter (10 trials per block). For each condition, three blocks of 288 trials were presented in succession, summing to a total of 714 standard and 150 deviant (17%) trials. Standards and deviants were pseudo-randomized in each block: at least three standards were separating two successive deviants and deviants could not occur in the first two trials of a block. The order of the conditions was pseudorandomized and counterbalanced, with one of the crossmodal conditions always being presented first.

### EEG Data Recording and Analysis

The Biosemi Active Two system (Biosemi, Amsterdam, Netherlands) was used for EEG data recording. EEG was measured from 64 active-channels, placed according to the 10–20 international system (Electro-cap International Inc., Eaton, OH, USA). The CMS electrode was used as the recording reference and was placed at the approximate location of PO1 (the DRL electrode was placed at the approximate location of PO2). Four additional Flat–Type Active electrodes of which two were placed below and above the left eye and two at the outer canthi of each eye were used to measure eye-movements. Two additional electrodes were placed on the right and left mastoids and used for offline re-referencing. Sampling rate of recording was 1024 Hz with a DC-104 Hz bandwidth. The offset range of the electrodes was kept between −20–20 mV.

The first two trials of each block and the first trial after each deviant were excluded from the analysis. Additionally, the first two trials after catch trials in the audiovisual conditions were not included in the analysis to avoid movement artifacts due to button presses (Luck, 2005). For each subject we randomly selected 150 of the remaining 480 standard trials for the further analysis, together with the 150 deviant trials. Analysis was performed with the EEGLAB toolbox (v11.0.0b; Delorme and Makeig, 2004)^1^ and MATLAB 2014a, (The MathWorks, Natick, MA, USA). We rereferenced data offline to the average of the left and right mastoids. Data was further bandpass filtered (1–70 Hz) and downsampled to 256 Hz. EEG epochs were extracted with respect to the onset of the auditory stimulus in all conditions, corresponding to epochs of −500 to 1200 ms in the Auditory and AV0 conditions and of −700 to 1000 ms in the AV200 condition. Baseline correction was performed with respect to the mean signal in the 500 ms baseline period prior to stimulation corresponding to −500 to 0 ms in the Auditory and AV0 conditions and −700 to −200 ms in the AV200 condition, thereby avoiding activation due to the letter in the AV200 in the baseline period. For visualization purposes all ERPs are shown for −500 to 1000 ms with respect to the auditory onset. We used a two-step protocol for artifact removal. First, we manually rejected the epochs containing non-stereotypical artifacts (e.g., electrode cable movements, rare jaw clinching). See Table 2 for the mean (SD) number of epochs included in the averages for the standards and deviants after this initial artifact rejection step. In the second step, we employed extended INFOMAX ICA (Lee et al., 1999) on 64 scalp channels resulting in 64 components per condition per participant. Independent component analysis (ICA) is optimal for identifying repeating stereotypical signals, including, for example, artifacts such as eye blinks, eye movements and muscle artifacts (e.g., swallowing) that constitute the major source of noise in EEG data of children. Independent components (ICs) were categorized as EEG activity or stereotypical non-brain artifacts based on visual inspection of their characteristic scalp topographies, time courses and power-frequency spectra (Jung et al., 2000). Components were classified as EEG activity according to the following criteria: (1) scalp topography indicating an underlying dipolar source; (2) spectral peaks typical of EEG; and (3) a regular occurrence across single trials (Delorme and Makeig, 2004). Independent components (ICs) representing non-brain artifacts were removed, and EEG data were reconstructed from the remaining component activations. See Table 2 for the mean (SD) number of components per subject in the different conditions. Single electrodes containing high amplitude noise were interpolated using spherical interpolation after the data was reconstructed. The reconstructed data was baseline corrected and low pass filtered at 30 Hz. Event related potentials (ERPs) were calculated for each participant by averaging the epochs per stimulus and condition.

**Table 2:** **Average number [M (SD)] of retained EEG independent components (ICs) and event trials for standard (S) and deviant (D) stimuli per condition**.

			Auditory	AV0	AV200
Number of retained ICs	T1		44 (7)	45 (7)	45 (7)
	T2		42 (6)	46 (6)	45 (8)
Number of retained trials	T1	S	148 (4)	149 (1)	147 (2)
		D	148 (5)	148 (1)	147 (3)
	T2	S	148 (2)	148 (3)	148 (2)
		D	148 (2)	147 (2)	148 (3)

We analyzed ERPs evoked by standard and deviant vowel sounds in the auditory and both audiovisual conditions at each of our measurements sessions to evaluate the presence of the MMN and LN. As we aimed to study how these auditory mismatch responses are influenced by the presentation of visual letters, we focused our analysis on fronto-central sites at which these responses are known to be maximal (Kujala et al., 2006; Bishop, 2007; Näätänen et al., 2007; Neuhoff et al., 2012). In particular, we included seven electrodes, Fz, FCz, Cz, F3, F4, FC3, FC4, covering the maximal MMN and LN responses in the auditory and crossmodal conditions at both measurement times. Thus, maximal MMN responses occurred at FCz (Auditory, AV0 at T1, Auditory, AV0, AV200 at T2) and FC3 (AV200 at T1) and maximal LN responses occurred at FCz (AV at T2), FC2 (Av200 at T1), Fz (AV0 at T1 and Auditory at T2), Cz (Auditory at T1) and F4 (AV200 at T2). Note that an analysis including the four fronto-central electrodes used in our previous study (Žarić et al., 2014), yielded the same pattern of results as the analysis including these seven electrodes. Individual MMN and LN peak latencies were manually determined within their respective time windows: 100–250 ms for the MMN and 600–750 ms for the LN (Froyen et al., 2009, 2011; Žarić et al., 2014). Mean amplitude across 50 ms centered on the individual peak latencies was used as the amplitude measure of the MMN and LN responses. Using SPSS, Version 21.0 (IBM Corp., Armonk, NY, USA), we first applied repeated measures ANOVAs with stimulus (two levels: standard and deviant) and electrode (seven levels) as within subjects factors per time window, per condition per measurement, to evaluate the presence of the MMN and LN responses. We subtracted the ERPs evoked by standards from those of deviants to obtain difference waves used in the analysis of crossmodal effects. We first performed repeated-measures ANOVA with condition (three levels: Au, AV0, AV200) and electrode (seven levels) as within subjects factors for the first measurement (T1) with the aim of testing whether the pattern of auditory and crossmodal ERP effects as previously observed in a larger group of 36 dyslexic children that participated at T1 (Žarić et al., 2014) would be preserved in the current subsample of 17 dyslexic children that participated at both T1 and T2. We then also performed this analysis at T2. In case of significant condition effects, we performed *post hoc* analysis of MMN/LN amplitude and latency measures on pairs of conditions (Au-AV0; Au-AV200) with condition (two levels) and electrode (seven levels) as within subject factors. Furthermore, we compared the amplitudes and latencies at the two measurements by including the within subject factors test (two levels: T1 and T2), condition (amplitude, three levels: Au, AV0, AV200; latency, two levels: AV0, AV200) and electrode (seven levels) in the repeated measures ANOVA. We report Greenhouse-Geisser corrected *p*-values for the factors for which the assumption of sphericity was violated. In addition to significance values, as in our previous study (Žarić et al., 2014) we report effect sizes as represented by partial eta squared (η_*p*_^2^) as this measure is more comparable between studies than classical eta squared (Lakens, 2013). In a further analysis, following our previous findings (Žarić et al., 2014), we analyzed the N1 and P2 responses to standard and deviant stimuli in the synchronous crossmodal condition (AV0) using repeated measures ANOVAs with stimulus (two levels: standard, deviant) and electrode (seven levels) as within subject factors. All *post hoc* tests were corrected for multiple comparisons by Bonferroni correction. Correction was employed for the two condition comparisons investigating crossmodal enhancement as compared to the auditory condition: AV0 vs. Auditory and AV200 vs. Auditory, resulting in a corrected α_Bonferroni_ = 0.025.

We employed linear regression to investigate how individual differences in ERP correlates of letter-sound associations relate to behavioral measures of reading fluency, reading accuracy and letter-speech sound coupling. To test replicability of the results from the first study (Žarić et al., 2014), we regressed MMN latency in the synchronous (AV0) condition on behavioral scores during the first measurement (T1). To test predictive power of this MMN latency on later behavioral scores, we analyzed its relation with behavioral measures from the second measurement (T2) as well as with behavioral gains from T1 to T2. Furthermore, as the LN latency in the crossmodal conditions significantly decreased from T1 to T2, also the relation between these measures and behavioral measures at T2 was analyzed. For linear regression, ERP latencies were computed as composite scores across seven fronto-central electrodes. Following our previous study behavioral measures were quantified in terms of composite scores for reading fluency (3DM word reading tests, the EMT, and “De Kat”), reading accuracy (3DM), letter-speech sound coupling accuracy (spelling, letter-speech sound identification and discrimination,) and speed (spelling, letter-speech sound identification and discrimination; Vaessen et al., 2010; Žarić et al., 2014). To examine specific relations between latencies and behavior we used simple linear regressions with one of the ERP latencies as a predictor and one of the behavioral composite scores as a dependent variable. We used false discovery rate (FDR) correction (Benjamini and Hochberg, 1995), as we conducted 12 tests for MMN AV0 latency and 8 tests for LN AV0 and AV200 latencies and report *p* values relative to *q*_(FDR)_ < 0.05 thresholds. Finally, to partial out autoregressive effects when testing MMN latency at T1 and reading fluency at T2, we employed linear regression with both MMN latency and reading fluency at T1 as predictors.

## Results

### Behavioral Tests

A comparison of behavioral test scores in the dyslexic children at the first (T1) and second (T2) measurements showed significant improvements at the group level on the raw scores of all tests, except for letter-speech sound identification and discrimination accuracies (Table 1). Moreover, results showed significant group-level increases in age-normed scores on the 3DM word reading tests (fluency and accuracy), as well as on 3DM spelling accuracy, demonstrating a greater improvement than would be expected just based on the time elapsed. At the group level both word reading fluency and accuracy were significantly impaired at T1 (below the 10th percentile, t-score of 37), while at T2 word reading accuracy reached the non-impaired range. At the level of individual children, word reading fluency improved with time and in 5 out of 17 children reached the non-impaired range at T2 (above the 10th percentile, t-score of 37). Reading accuracy scores also improved with 6 children scoring in the non-impaired range at T1 and 10 children at T2. Note that this pattern of results is consistent with the finding that in relatively shallow orthographies such as Dutch, reading fluency is the most persistent deficit in dyslexia (Ziegler et al., 2003; Wimmer and Schurz, 2010; Blomert, 2011).

### Auditory MMN and LN Effects

**Figure 2:**
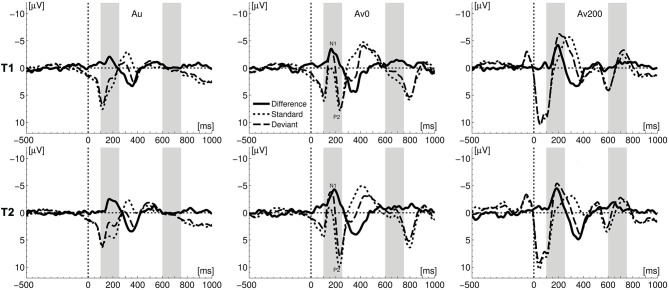
**Comparison of ERPs for deviant and standard stimuli at the two measurements (T1 and T2).** Light gray bars visualize the mismatch negativity (MMN) and late negativity (LN) windows of interest; Au—auditory, AV0 simultaneous crossmodal, AV200 asynchronous crossmodal condition.

**Figure 3:**
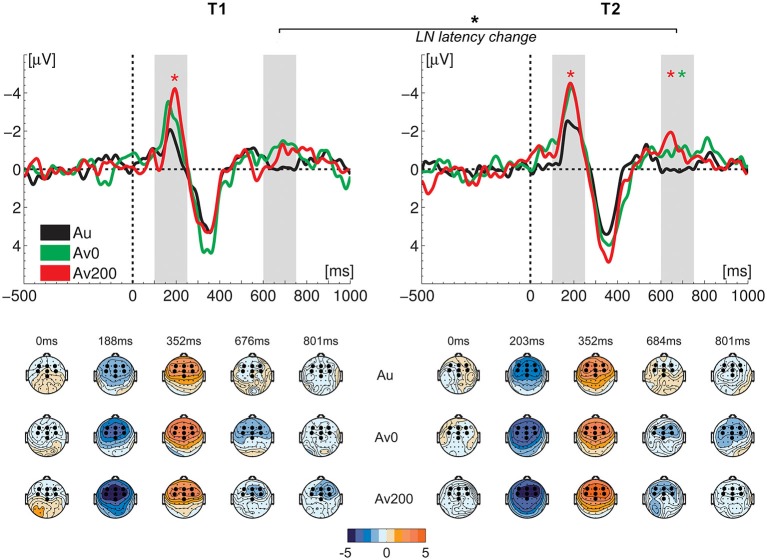
**Difference waves (upper row) and topographical maps (lower row) at the two measurement times (T1 and T2).** Light gray bars visualize the MMN and LN time windows of interest; green and red asterisks indicate a significant MMN/LN amplitude enhancement in respectively the synchronous (AV0) and asynchronous (AV200) crossmodal conditions relative to the auditory condition (Au); black asterisk indicates a significant crossmodal LN latency difference from T1 to T2. Amplitude measures did not show significant differences from T1 to T2. Black hexagons in topographical maps represent the seven fronto-central electrodes used in the analysis.

We first analyzed the MMN and LN responses evoked in the auditory condition as they served as a baseline for crossmodal enhancement effects, At both measurement times, the vowel deviant elicited an auditory MMN (Figure 2) with the expected fronto-central topographical distribution (Figure 3), leading to main effects of stimulus at T1 (*F*_(1,16)_ = 20.93, *p* < 0.001, η_p_^2^ = 0.567) and T2 (*F*_(1,16)_ = 47.59, *p* < 0.001, η^2^ = 0.748). At both measurement times, an auditory LN response was elicited around 670–685 ms after stimulus onset with main effects of stimulus at T1 (*F*_(1,16)_ = 15.22, *p* = 0.001, η_p_^2^ = 0.488) and T2 (*F*_(1,16)_ = 11.82, *p* = 0.003, η_p_^2^ = 0.425). A comparison of auditory MMN and LN responses measured at T1 and T2, showed no significant changes between measurement times in terms of response amplitude or latency.

### Letter-Effects: Crossmodal MMN and LN Amplitudes

The deviant vowel sound /o/ elicited MMN and LN responses in both crossmodal conditions (Figures 2 and 3). One crucial quantification of actual crossmodal processing involves testing the effect of the double violation due to the presentation of the letter “a” in the crossmodal conditions. In other words, did the deviancy of the vowel sound /o/ from both the standard vowel sound /a/ and the letter “a” significantly enhance the MMN and/or LN responses as compared to the auditory only condition (Froyen et al., 2008, 2009; Žarić et al., 2014). To this end, we performed repeated measures ANOVAs on average MMN and LN amplitudes extracted from ERP difference waves over seven fronto-central electrodes, with condition (auditory, AV0, AV200) and electrode (seven levels) as within-subject factors.

We first compared the auditory and crossmodal conditions at T1 to test the pattern of amplitude enhancements in the smaller sample of dyslexic children that participated both at T1 and T2 as compared to a larger sample that only participated at T1 (Žarić et al., 2014). In the MMN window, this analysis yielded a main effect of condition (*F*_(2,32)_ = 3.93, *p* = 0.030, η_p_^2^ = 0.197). A comparison of crossmodal conditions with the auditory condition revealed a significant crossmodal enhancement in the asynchronous AV200 condition (*F*_(1,16)_ = 9.64, *p* = 0.007, η_p_^2^ = 0.376) while this enhancement did not reach significance in the simultaneous AV0 condition (*F*_(1,16)_ = 4.62, *p* = 0.047, η_p_^2^ = 0.224) after Bonferroni correction (α = 0.025). In the LN window, dyslexic children showed no significant crossmodal enhancement (main effect of condition (*F*_(2,32)_ = 1.26, *p* = 0.297, η_p_^2^ = 0.073). This pattern of a significant crossmodal MMN enhancement in the AV200 condition, together with the absence of this enhancement in the AV0 condition and of later LN enhancements in both conditions, resembles the pattern of results of the most severely dysfluent children in a larger sample of dyslexics (Žarić et al., 2014). In particular, in this larger group of dyslexics, the most severely dysfluent children, did not show a significant MMN enhancement in the AV0 condition. Ten of the current 17 children belonged to this severely dysfluent group, which explains the absence of a significant MMN enhancement in this condition.

As our previous results showed that the reduced crossmodal MMN response in the AV0 condition in severely dysfluent dyslexics was due to a significant letter-speech sound deviancy effect in the N1 but not in the P2 window, we also separately analyzed these two responses. Results showed the same pattern of results in the current subsample at T1, with a significant crossmodal deviancy effect in the N1 (*F*_(1,16)_ = 20.45, *p* < 0.001, η_p_^2^ = 0.561), but not in the P2 window.

An analysis of MMN and LN enhancements measured 6 months later, at T2, yielded a marginally significant effect of condition in the MMN window (*F*_(2,32)_ = 3.23, *p* = 0.052, η_p_^2^ = 0.168). A comparison of separate conditions showed that this effect was due to a significant crossmodal MMN enhancement in the AV200 condition (*F*_(1,16)_ = 7.39, *p* = 0.015, η_p_^2^ = 0.316) but not in the AV0 condition. In contrast to T1, at T2, a significant main effect of condition was observed in the LN window (*F*_(2,32)_ = 7.18, *p* = 0.003, η_p_^2^ = 0.310). The crossmodal LN response was significantly enhanced in both the AV0 (*F*_(1,16)_ = 7.09, *p* = 0.017, η_p_^2^ = 0.307) and AV200 (*F*_(1,16)_ = 13.34, *p* = 0.002, η_p_^2^ = 0.455) conditions as compared to the auditory condition (*α*_Bonferroni_ = 0.025). A direct comparison of the two measurements did not yield significant differences between T1 and T2 for either the MMN or the LN response amplitudes. That is, the ANOVA with test (two levels), condition (three levels) and electrode (seven levels) as repeated measures did not yield significant main effects of test or test*condition interactions for the MMN/LN amplitudes. However, unlike at T1 (Žarić et al., 2014), this overall analysis did yield significant main effects of condition for both the MMN (*F*_(2,32)_ = 10.95, *p* < 0.001, η_p_^2^ = 0.406) and LN (*F*_(2,32)_ = 7.55, *p* = 0.002, η_p_^2^ = 0.321) amplitudes.

A separate analysis of N1 and P2 deviancy effects at T2 showed a significant mismatch response (*α_Bonferroni_* = 0.025) in both the N1 (*F*_(1,16)_ = 14.10, *p* = 0.002, η_p_^2^ = 0.468) and P2 windows (*F*_(1,16)_ = 9.08, *p* = 0.008, η_p_^2^ = 0.362). There was a tendency for an increase in the mismatch response from T1 to T2 in the P2 window (test: *F*_(1,16)_ = 3.09, *p* = 0.098, η_p_^2^ = 0.162; stimulus: *F*_(1,16)_ = 9.71, *p* = 0.007, η_p_^2^ = 0.378), while there was no difference between T1 and T2 in the N1 window (stimulus: *F*_(1,16)_ = 34.93, *p* < 0.001, η_p_^2^ = 0.686).

### Letter Effects: MMN and LN Latency

The timing of the letter effects was investigated by analysis of MMN and LN latencies in both crossmodal conditions. The ANOVA with test (two levels), condition (two levels) and electrode (two levels) as repeated measures yielded no difference in the timing of the crossmodal MMN responses between measurement times. Most interestingly, we did observe a shortening of the LN latency from T1 to T2 (*F*_(1,16)_ = 7.37, *p* = 0.015, η_p_^2^ = 0.315) without a significant main effect of condition or test*condition interaction.

### ERP—Behavior Relations

**Figure 4:**
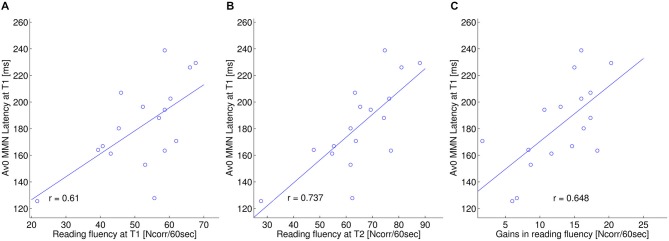
**ERP—behavior relation: MMN window.** Relation between the MMN latency (ms) in the simultaneous crossmodal condition (AV0) at T1 and: **(A)** reading fluency (composite score including: 3DM, EMT, “De kat”; Ncorr. words/60 s) at T1; **(B)** reading fluency at T2; **(C)** gains in reading fluency from T1 to T2.

**Figure 5:**
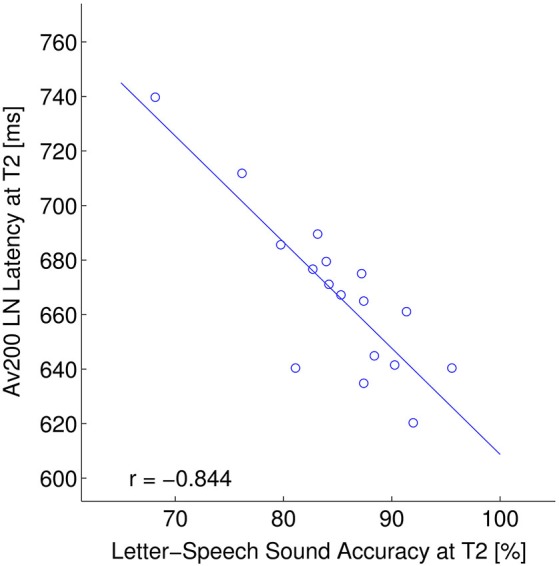
**ERP—behavior relation: LN window.** Relation between the LN latency (ms) in the asynchronous crossmodal condition (AV200) and letter-speech sound accuracy (composite score including: spelling, letter-speech sound identification and letter-speech sound discrimination accuracy) (% correct) at T2.

In a further analysis we investigated how (changes in) MMN and LN measures relate to off-line behavioral measures of reading related skills. First, similar to our previous study (Žarić et al., 2014), at T1 we regressed the latency of the MMN in the AV0 condition with composite behavioral scores, including reading fluency and accuracy as well as letter-speech sound coupling accuracy and speed (Figure 4). Second, we investigated the predictive value of the MMN latency in the AV0 condition on later behavioral performance. And finally, we asessed the relation between behavioral performance and LN latency (Figure 5). We only report FDR corrected significance values (*q* < 0.05).

As for the correlations between MMN latency in the AV0 condition and behavioral scores at T1, also in the current sample of dyslexics MMN latency significantly correlated with individual differences in word reading fluency (*r* = 0.610; *p* = 0.009; Figure 4). Moreover, the latency of the MMN in the AV0 condition at T1 was predictive of word reading fluency at T2 (*r* = 0.737, *p* = 0.001) and of gains in word reading fluency from T1 to T2 (*r* = 0.648, *p* = 0.005). Furthermore, MMN latency at T1 was a significant predictor of reading fluency at T2 even when the autoregressive effects of reading fluency at T1 were removed. Thus, linear regression including both MMN latency and reading fluency at T1 as predictors yielded a significant overall effect (*r* = 0.962, *p* < 0.001) as well as separate effects for MMN latency (*r_partial_* = 0.603, *p* = 0.013) and reading fluency at T1 (*r_partial_* = 0.914, *p* < 0.001). In all cases and in accordance with our first study, a longer MMN latency was associated with better performance.

As the crossmodal LN latency significantly changed following the training, we also tested whether this change relates to behavioral scores. AV200 LN latency at T2 regressed significantly on composite scores of letter-speech sound accuracy (*r* = −0.844, *p* < 0.001) at T2 (Figure 5). LN latency in AV0 condition did not significantly correlate with behavioral measures after correction for multiple comparisons.

## Discussion

The present study investigated the development of ERP measures of letter-speech sound integration and speech processing in dyslexic children over a period of 6 months during which they received both their regular third grade reading curriculum and extensive training in letter-speech sound coupling (Tijms, 2007). We were interested whether reduced crossmodal ERP responses in dyslexics show any indication of malleability and whether ERP changes relate to behavioral improvements. To this end we investigated crossmodal influences on vowel evoked MMN and LN responses due to the presentation of the letter “a” together with the standard vowel sound “a” and the deviant vowel sound “o” (Froyen et al., 2008, 2009, 2011; Žarić et al., 2014). Our results of the first measurement (T1) demonstrate normal auditory MMN/LN change detection responses to spoken vowels /a/ and /o/ together with reduced letter-speech sound integration, thereby confirming previous findings in the current subsample of dyslexic children (Žarić et al., 2014). They also confirm a significant relation between individual differences in reading fluency and the latency of the MMN in the simultaneous crossmodal condition (AV0). The present study extends these findings by showing that the AV0 MMN latency also predicts reading fluency at the second measurement (T2) and gains in reading fluency from T1 to T2. Interestingly, our results suggest moderate improvements in the neural integration of letters and speech sounds over a 6 months period including schooling and training, with earlier (and enhanced) crossmodal effects in the LN window. Furthermore, earlier LN latency at T2 correlated significantly with higher behavioral accuracy in letter-speech sound coupling.

Auditory deviancy elicited significant MMN and LN responses with a typical fronto-central topographical distribution at both measurements, confirming typical MMN change detection responses to the vowels in children with developmental dyslexia (Czamara et al., 2011; Froyen et al., 2011; Roeske et al., 2011; Neuhoff et al., 2012; Žarić et al., 2014). In fact, our crossmodal paradigm included vowels as this allows the investigation of letter-speech sound integration deficits beyond possible speech processing deficits that have been found using more subtle speech changes involving, for example stop consonants (e.g., Schulte-Körne et al., 1998; Csépe, 2003). Correspondingly, the vowel-evoked MMN and LN responses did not change significantly following the training. This is in concordance with the results of a behavioral study which showed that pre-reading children at familial risk for dyslexia may exhibit problems in learning letter–sound associations during a training without phonological deficits before or after the training (Blomert and Willems, 2010). As for the general pattern of auditory evoked responses, the relatively small N1-P2 complex in the auditory as compared to the AV0 condition is in line with previous findings using the same paradigm in typical readers of different age. That is, a comparably small N1-P2 response was observed in the auditory condition in 8-year-old children, with a more pronounced response in 11-year-old children (Froyen et al., 2009) and a clear response in adults (Froyen et al., 2008). Furthermore, in developmental ERP studies using e.g., click sounds or speech sounds it is often not possible to measure a clear N1 and/or P2 response in children younger than 10 years of age (see e.g., Ponton et al., 2000; Maurer et al., 2003a; Bonte et al., 2007). Although this would need to be addressed in future research, one may speculate that the clear N1-P2 response elicited by standard and deviant stimuli in the AV0 condition is due to an enhancement of auditory responses when accompanied by simultaneous visual stimuli (see e.g., Giard and Peronnet, 1999; Fort et al., 2002a,b; Hyde et al., 2010).

### Reduced LN Letter Effects and Changes in its Neural Timing

In 9-year-old normally reading children we previously observed enhanced MMN and LN responses to both synchronous and asynchronous letter-speech sound pairs (Žarić et al., 2014). Similar to the larger sample of 36 9-year-old dyslexic children that participated at T1 (Žarić et al., 2014), the present subsample of 17 dyslexic children that participated both at T1 and T2, initially showed no LN enhancement due to the presentation of the letter, confirming weak neural integration of letters and speech sounds (Blau et al., 2010; Žarić et al., 2014). The children did show a crossmodal MMN enhancement, although, like in the most severely dysfluent dyslexics of our previous study (Žarić et al., 2014), this effect only reached significance in the AV200 condition. A reduced LN in response to the deviant speech syllable /ba/ relative to the standard syllable /da/, in a broader time window (300–700 ms) overlapping with our crossmodal LN (600–750 ms), has been proposed as a potential endophenotype for dyslexia (Czamara et al., 2011; Roeske et al., 2011; Neuhoff et al., 2012). Although the functional role of the LN is not yet well understood, with respect to more automatic, perceptually driven letter-speech sound integration in the MMN window, the crossmodal LN enhancement may reflect more cognitive, associative and/or attentional aspects of this integration. In particular, multiple studies using different paradigms have indicated a late sensitivity to orthographic-phonological interactions in children (Froyen et al., 2009; Hasko et al., 2013; Jost et al., 2013; Žarić et al., 2014) and adults (Pattamadilok et al., 2011; Savill and Thierry, 2011; Lafontaine et al., 2012). While these late orthographic-phonological interactions are typically observed during demanding meta-phonological tasks in adults (Pattamadilok et al., 2011; Lafontaine et al., 2012), in children they may also occur during the integration of simple letter-vowel pairs (Froyen et al., 2009; Žarić et al., 2014), and integration of audiovisual words (Jost et al., 2013). These late effects were found to be disrupted in dyslexic children (Froyen et al., 2011; Hasko et al., 2013; Žarić et al., 2014) and adults (Savill and Thierry, 2011). Together with these previous findings, the observed reduced crossmodal LN effect may thus suggest a diminished capability to access and/or manipulate letter-speech sound representations (Blau et al., 2010; Savill and Thierry, 2011; Ramus and Ahissar, 2012), or impairments in attentionally-mediated integration (Czamara et al., 2011; Savill and Thierry, 2011, 2012; Neuhoff et al., 2012) in dyslexic children. It still remains an open question whether this impaired letter-speech sound integration is a specific deficit, or whether it stems from a more fundamental impairment in audiovisual integration (Hahn et al., 2014).

Whereas in both crossmodal conditions, the LN effect did reach significance at the second measurement, the size of this effect did not change significantly from T1 to T2. Significant changes in letter-speech sound integration were only observed in the neural timing of this effect. That is, LN latency in the crossmodal conditions did show a significant decrease from T1 to T2. The behavioral relevance of this change is further indicated by the observation that earlier LN latency at T2 significantly related to more accurate behavioral coupling of letters and speech sounds. This earlier LN at T2 may thus be a sign of an improvement in the capability to access and/or manipulate the crossmodal representations (Blau et al., 2010; Savill and Thierry, 2011; Ramus and Ahissar, 2012). In future studies, it would be interesting to test whether these type of ERP latency changes relate to observed increases in white matter connectivity following behavioral intervention in 8–10-year-old poor readers (Keller and Just, 2009). Although the crossmodal LN latency related to behavioral letter-speech sound coupling at T2, we measured only one group of children that all followed the same training, which makes it difficult to fully disentangle specific training effects and more general maturational changes. On the other hand, if the shortening of crossmodal LN latency were fully determined by maturational changes in ERP morphology and/or timing, we would expect similar changes in the auditory only condition, which was not the case (Shafer et al., 2000; Martin et al., 2003; Maurer et al., 2003b). Furthermore, although latency measures of sustained ERP responses such as the LN may show larger intersubject variability as compared to shorter-lasting responses such as the MMN, this appears not to be the case in the present data as the range of individual participant’s peak latencies was comparable across the MMN and LN responses (for example see Figures 4 and 5). Together, the present findings suggest moderate improvement in the neural integration of letters and speech sounds due to a combination of intensive training together with the regular curriculum, especially in the later (timing) aspects of this integration.

### Reading Fluency and the Timing of the Crossmodal MMN

In addition to a potentially malleable late integration of letters and speech sounds, our results also suggest a less flexible early integration that may form a bottleneck in developing reading fluency. As in our previous study (Žarić et al., 2014), in the present subsample of 9-year-old dyslexic children, better reading fluency significantly correlated with a longer latency of the MMN in the simultaneous crossmodal condition (AV0). In particular, AV0 MMN latency not only correlated with reading fluency at T1, but also with reading fluency at T2 and with fluency gains from T1 to T2. Based on a further analysis of the underlying N1 and P2 ERP components, our previous results showed that this was due to a shorter-lasting, reduced MMN response, encompassing only the N1 window in a group of most severely dysfluent dyslexics as compared to a longer lasting MMN response, encompassing both the N1 and P2 windows in moderately dysfluent dyslexic and typical readers (Žarić et al., 2014). As 10 of the current 17 children belonged to the severely dysfluent group, at the group level our results at T1 showed a shorter-lasting, reduced mismatch response in the AV0 condition, with a significant crossmodal enhancement in the N1 but not the P2 window (and no significant crossmodal MMN enhancement). Although little is known on the specific functional processes underlying the P2, recent evidence indicates that this deflection is specifically sensitive to the audiovisual integration of orthographic and phonological units (Pattamadilok et al., 2011) and of visual articulatory gestures and speech (van Wassenhove et al., 2005; Pilling, 2009; Baart et al., 2014; Knowland et al., 2014). The N1 window may thus reflect a first step of audiovisual convergence, independently of reading fluency, which, especially in the most severely dysfluent dyslexics is followed by a reduced speech specific integration of letters and speech sounds in the P2 window (Žarić et al., 2014).

The absence of a significant crossmodal MMN in the synchronous condition may represent difficulties in forming a clear letter-speech sound representation (Blomert, 2011), like “graphonemes” (Whitney and Cornelissen, 2005). This more basic impairment, usually associated with severe cases (Roeske et al., 2011; Žarić et al., 2014), could be more resistant to change, than later more cognitive processes. In agreement with this suggestion, neither the latency nor the amplitude of the MMN letter effect showed a significant change from T1 to T2. Moreover, the children with later AV0 MMN latency (i.e., longer lasting MMN responses) at the time of the first measurement: (1) read more fluently at the beginning; (2) stayed more fluent at second measurement; and (3) gained more from literacy instruction and training. Although further studies are needed, the timing of this type of crossmodal change detection responses may provide a biomarker that could contribute to a better prediction of reading gains and/or individual tailoring of dyslexia training/intervention strategies (Leppänen, 2013).

### Conclusion

Learning to read and its first step of mapping letters to speech sounds influences cortical networks underlying visual, auditory and higher-order language functions (Brem et al., 2010; Dehaene et al., 2010). Failure to successfully map letter-speech sound correspondences may be a proximate cause of reading failure (Blau et al., 2010; Blomert, 2011) and is related to an attenuated brain activation in dyslexia (Blau et al., 2010; Froyen et al., 2011; Žarić et al., 2014). The present findings suggest that the reduced neural integration of letters and speech sounds in dyslexic children may show moderate improvement over a period with reading instruction and letter-speech sound training, particularly in the timing of later aspects of this integration (LN window). Our findings additionally point to a less flexible early integration (MMN window), with individual differences in its timing predicting gains in reading fluency. Further research should examine these improvements on a longer time scale as it is a “long road” to fully automatized letter-speech sound integration (Froyen et al., 2008, 2009). Moreover, it would be important to further differentiate effects specific to intensive training in letter-speech sound coupling and more general maturational effects, by additionally following longitudinal trajectories of letter-speech sound integration in dyslexic children that do not receive this training. Together these studies will contribute to a better prediction of reading gains and/or individual tailoring of dyslexia training/intervention strategies.

## Conflict of Interest Statement

The authors declare that the research was conducted in the absence of any commercial or financial relationships that could be construed as a potential conflict of interest.

## Footnotes

^1^http://www.sccn.ucsd.edu/(eeglab)
